# Effect of Cold Adaptation on the State of Cardiovascular System and Cardiac Tolerance to Ischemia/Reperfusion Injury

**DOI:** 10.61186/ibj.3872

**Published:** 2023-08-09

**Authors:** Nikita S. Voronkov, Sergey V. Popov, Natalia V. Naryzhnaya, N. Rajendra Prasad, Ivan M. Petrov, Viktor V. Kolpakov, Evgenia A. Tomilova, Ekaterina V. Sapozhenkova, Leonid N. Maslov

**Affiliations:** 1 Cardiology Research Institute, Tomsk National Research Medical Center of the RAS, Tomsk, Russia;; 2 Department of Physiology, Tomsk State University, Tomsk, Russia;; 3 Department of Biochemistry and Biotechnology, Faculty of Science, Annamalai University, Annamalainagar, Tamilnadu, India;; 4 Tyumen State Medical University, Tyumen, Russia

**Keywords:** Acclimatization, Cold temperature, Heart, Ischemia, Reperfusion

## Abstract

Despite the unconditional success achieved in the treatment and prevention of AMI over the past 40 years, mortality in this disease remains high. Hence, it is necessary to develop novel drugs with mechanism of action different from those currently used in clinical practices. Studying the molecular mechanisms involved in the cardioprotective effect of adapting to cold could contribute to the development of drugs that increase cardiac tolerance to the impact of ischemia/reperfusion. An analysis of the published data shows that the long-term human stay in the Far North contributes to the occurrence of cardiovascular diseases. At the same time, chronic and continuous exposure to cold increases tolerance of the rat heart to ischemia/ reperfusion. It has been demonstrated that the cardioprotective effect of cold adaptation depends on the activation of ROS production, stimulation of the β_2_-adrenergic receptor and protein kinase C, MPT pore closing, and K_ATP_ channel.

## INTRODUCTION

Hospital mortality in patients with STEMI is 4.6%-7.5% , which has not decreased in recent years[1^-^4]. Moreover, drugs that have been approved for clinical use and are capable of preventing reperfusion injury of the heart with high efficacy are not currently available[5,6]. During recent years, the attention of investigators has greatly been drawn to the study of the molecular mechanisms of the cardioprotective effect of pre- and post-conditioning, believing that this knowledge contributes to the development of drugs that increase cardiac tolerance to reperfusion injury[7]. The study of the trigger and molecular mechanisms underlying the infarct-reducing effect of cold adaptation can contribute to the identification of molecular targets for developing novel cardioprotective drugs.

**Information sources**

The National Library of Medicine’s PubMed (https://pubmed.ncbi.nlm.nih.gov/) was searched to acquire information on the subject of the article. About 3000 abstracts were studied, and 1200 full-text articles on cold adaptation and cold exposure were identified. Also, 62 articles were found in Russian on cold adaptation and exposure in Russian libraries. The duration of searching was about six months (from April 2022 to October 2022), and a total number of 172 papers were included in this review. 

**Cold and human**

**Effect of cold environment on the state of the cardiovascular system in the Far North population**

The health of people who came to work in Norilsk and Dikson, cities located above the Arctic Circle, was investigated in an earlier study[8]. In 1964, it was documented that healthy residents of Norilsk had higher BP than those living in Central Asia[9]. A persistent BP increase has been also observed in migrants living in the Far North. However, arterial hypertension was much less common in the indigenous population of the northern regions of Russia[^10^]. The incidence of hypertension among migrants in the Far North raises with increasing the length of residence in the Arctic area, reaching 61% in people living in this region for more than 15 years[11]. Moreover, a higher prevalence of hypertension was observed among the shift workers than the rest of the Russian population[12]. In addition, an increase in the incidence of AMI cases and the mortality rate from CVD was found among the newcomer population of the Far North, while in the indigenous inhabitants of this region who lead a traditional lifestyle, AMI was relatively rare[^10^]. The incidence of CHD in people aged 50-59 years living in the Arctic for less than 10 years was reported as 25%, but this rate increased to 45% for those who had been living in the Far North for more than 10 years (*p* < 0.001). Therefore, long-term residence in the Arctic is considered a risk factor for the occurrence of CHD[8]. At the same time, the incidence of CHD was lower among the indigenous population, leading a traditional lifestyle, than those residents of the middle latitudes of the Union of Soviet Socialist Republics[8]. According to Turchinskiǐ, aboriginals of the Arctic who have preserved the traditions and lifestyle of their ancestors, practically experienced no hypertension[8]. However, Yakuts living in the city of Yakutsk in the Arctic region had a high incidence of CHD and hypertension[13]. The incidence of AMI among migrants arriving in the Far North increased sharply after 7 to 10 years[8]. In Norilsk, 24% of AMI patients are comprised of young people aged less than 44 years old[14]. Mortality from CVD among the male population of Yakutsk aged 20-54 years is 38.4% of the total mortality[15], which is significantly higher than the rate reported in South/Middle Russia[16]. Atherosclerotic lesion of the aorta and atherosclerosis of the coronary arteries in Yakutsk are more common in the newcomers than in the indigenous population[15]. The incidence of CVD in the Siberian Federal District, compared to Russia, is also higher as a whole[17]. Melnikov[18] found that in Novosibirsk (a Southern Siberian city), the average age of individuals who died from CVD was 59 years old, and among the inhabitants of Mirny (Yakutia, Russia) and Yakutsk, this indicator was 52 and 55 years, respectively. Danish researchers have shown that CVD mortality among the Greenland population is two times higher than that of Danish people[19]. 

**Seasonal variations in morbidity and mortality of patients with CVD**

**Fig. 1:**
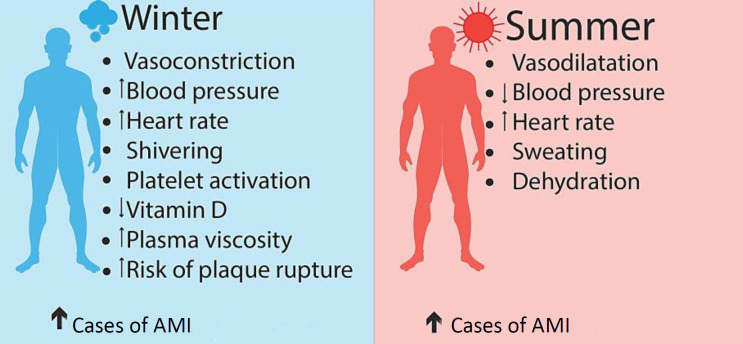
Reasons behind the increased AMI in cold and warm conditions

**Fig. 2:**
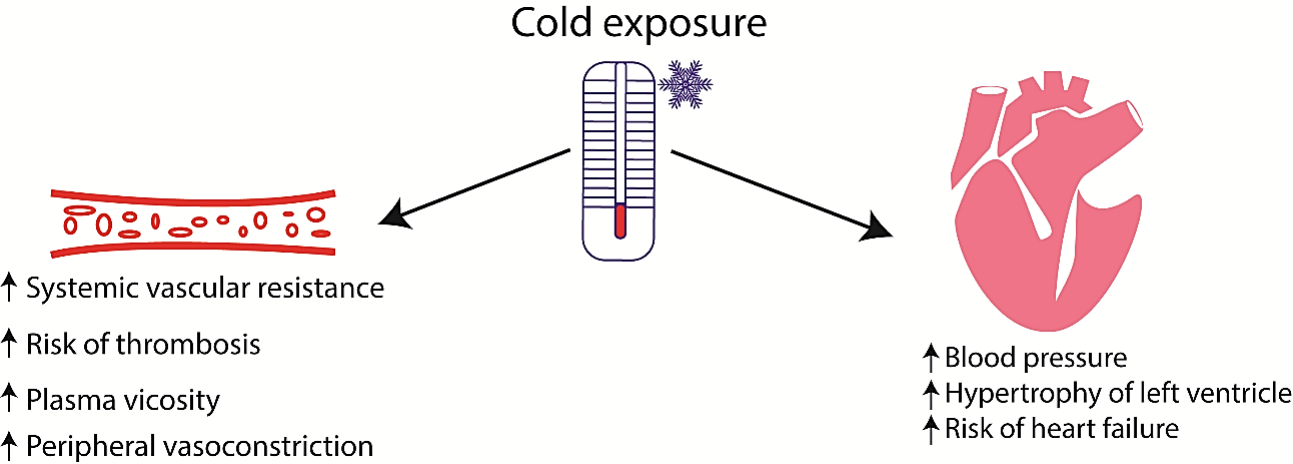
Blood circulation and heart and responses to cold exposure

Approximately 10% more cases of AMI were observed in winter or spring than in summer in Virginia^[^^20^^]^, and approximately 53% more AMI cases were reported in winter than in the summer in Massachusetts^[^^21^^]^. There was a negative correlation between hospital admissions of patients with acute coronary syndrome and mean daily temperature in Athens (Greece)[22]. In Hungary, a peak period of the incidence of AMI was found during spring[23], while the minimum number of events was recorded during summer. This pattern was also identified in Germany, London (UK), Yekaterinburg (Russia), Northern Ireland, and Finland[24-28]. According to Barnett et al., in cold periods, the rate of coronary event increases more in populations living in warm climates than those living in cold climates[29]. High ambient temperatures can also increase mortality from CVD (Fig. 1)[30]. Increased AMI morbidity and mortality during the cold season are associated with the activation of the adrenergic system[31], an elevation in blood viscosity, and an enhancement in platelet aggregation (Fig. 2)[32].

**Effects of cold on the cardiovascular system**

***Adverse effects of cold adaptation on the cardiovascular system***

One of the main negative effects of cold adaptation is hypertension. BP increases after prolonged exposure to cold in animals[33,34] and in humans (Fig. 2)[17,35]. In animals, when adapting to cold (1-4 ºC), cardiac hypertrophy develops[34]. There are also data on cardiac hypertrophy in humans during chronic cold exposure[35]. It has been observed that left ventricular hypertrophy develops after continuous cold exposure (4 °C; 4 weeks) without a change in the right ventricle weight[34]. Similarly, intermittent cold exposure (4 °C; 1.5 or 8 h daily; 4 weeks) did not induce cardiac hypertrophy[34].

Our data coincide with those of a research group in USA[^36^^-^^38^]. They found that long-term cold exposure (5 ± 2 ºC; 8 weeks) induced an increase in the left ventricular weight without alterations in the right ventricular weight. In addition, cardiac hypertrophy was reversible and disappeared four weeks after the cessation of cold exposure. Importantly, mild cold adaptation (8 ± 1 ºC; 5 weeks) did not affect the weight of the left ventricle[39]. All the above data clearly show that the adverse effects of cold adaptation depend on its severity, and thus the ambient temperature for adaptation should carefully be taken into account. 

***Role of aldosterone, angiotensin-II, and endothelins in the adverse effects of cold adaptation***

**Fig. 3:**
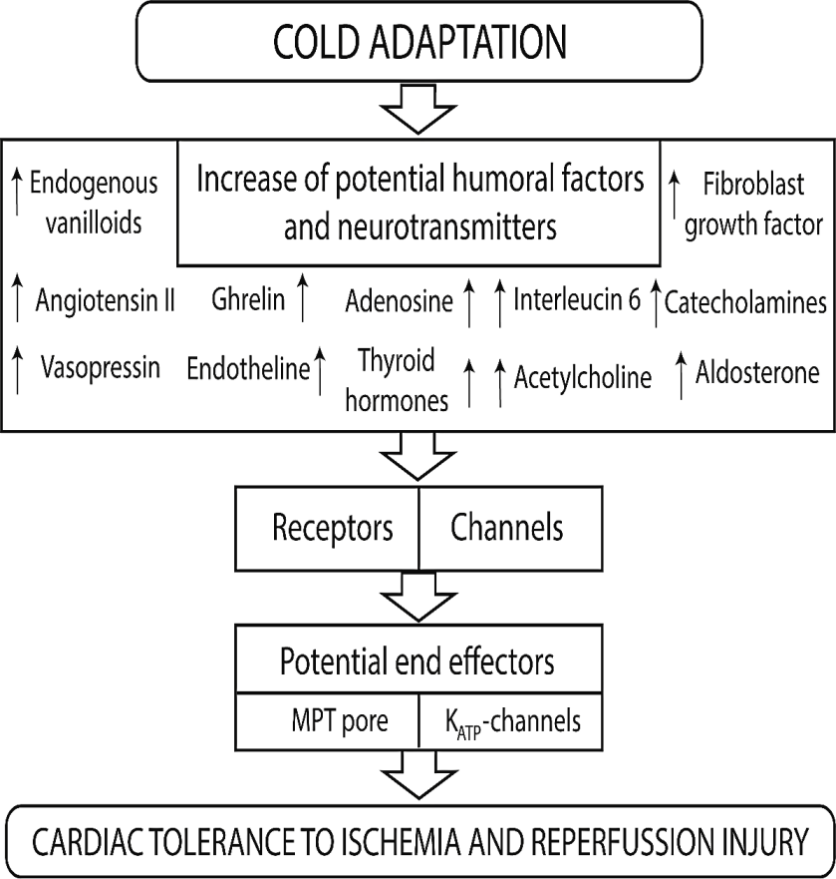
Potential signaling pathways of cardiac tolerance to IRI during cold adaptation

It has been documented that aldosterone, angiotensin-II, and endothelins play an important role in the development of hypertension. Moreover, they can be involved in the development of cardiac hypertrophy[40-45]. It has also been reported that a 17-day ski trip in the Far North at temperature ranging from -30 to -40 °C causes a two-fold elevation in the plasma aldosterone concentration[46]. However, some investigators were unable to detect an increase in the plasma aldosterone concentration in the rats following cold exposure (5 °C; 3 weeks)[38] and cold adaptation (4 °C; 14 days)[47], though the plasma aldosterone level increased after seven days of cold exposure (4 °C)[47]. Repeated cold water immersions (three times a week for six weeks) did not alter the plasma aldosterone concentration in male swimmers in winter[48], which is likely due to the fact that the exposure was not intense enough to induce an increase in the plasma aldosterone level. Repeated cold exposure (4 °C; 1 h daily; 19 days) induced an increase in plasma aldosterone concentration in rats[49]. Cold exposure (5 ± 2 °C; 4 weeks) also induced hypertension and cardiac hypertrophy in rats. Daily administration of spironolactone prevented the development of hypertension, but not cardiac hypertrophy[50]. Adenoviral delivery of renin antisense inhibited the development of hypertension after adaptation to cold (6.7 ± 2 °C; 1, 3, and 5 weeks) in rats[51]. The recombinant adeno-associated virus carrying short-hairpin small-interference RNA for the mineralocorticoid receptor was administered to mice during cold exposure (6.7 °C; 32 days)[33]. This adenoviral construct prevented a cold-induced increase in BP. The mentioned data indicate that aldosterone is involved in the development of cold-induced hypertension, but not cardiac hypertrophy. In 1993, Cassis found that cold exposure (4 °C; 7 days) had no effect on the plasma angiotensin II level in rats[52], but later in 1998, he and his colleagues discovered that cold exposure (4 °C; 7 days) led to an increase in the plasma concentration of angiotensin II in these animals[53]. Shechtman et al. also demonstrated that treatment with captopril could prevent cold-induced hypertension (5 ± 2 °C; 4 weeks) in rats, whereas it has no effect on cardiac hypertrophy[54]. Treatment with the AT_1_ receptor antagonist losartan was found to prevent cold-induced hypertension (5 ± 2 °C, 3 weeks) in rats but did not abolish the development of cardiac hypertrophy[55]. Pressor response to a bolus injection of angiotensin-II increased in cold-adapted rats (5 ± 2 °C; 3 and 4 weeks)[56]. These data were confirmed by other investigators who found that cold adaptation enhanced the responsiveness of tail arteries to angiotensin II in rats[57]. It was shown that cold exposure (5 °C; 5 weeks) did not increase BP in angiotensinogen gene-knockout mice[58]. The above-mentioned evidence convincingly shows that angiotensin-II is involved in the development of cold-induced hypertension through the activation of the AT_1_ receptor. ET-1 is a potent vasoconstrictor. It was demonstrated that cold exposure (6.7 ± 2 °C; 1, 3, and 5 weeks) increased BP and also ET-1 level in the heart and mesenteric arteries in rats^[^^59^^]^. However, investigators did not find any alteration in the concentration of ET-1 in plasma. In a study performed by Chen et al., cold exposure doubled the expression of ET_A_ receptor protein, while the expression of the ET_B_ receptor decreased by 90%, in the heart of cold-exposed rats. Cold exposure also increased the ET_A_/ET_B_ receptor ratio in the heart by about 60-fold[59]. In another investigation by Zhang et al., wild-type and ET_A_ receptor knockout mice were exposed to cold (4 °C) for 2 and 5 weeks. They found that cold adaptation induced severe cardiac fibrosis in wild-type mice, and ET_A_ receptor knockout abolished these negative manifestations of cold adaptation[60]. Consequently, endogenous ET-1 could be involved in cardiac fibrosis through the activation of ET_A_ receptors. Endogenous catecholamines do not seem to play a role in the development of cold-induced hypertension, as pressor response to a bolus injection of the α-AR agonist phenylephrine decreased in cold-adapted rats (5 ± 2 °C; 3 and 4 weeks)[56]. Chronic treatment with the α-AR antagonist prazosin had no effect on the development of cold-induced hypertension in rats (5 ± 2 °C; 3 and 4 weeks)[61]. As a result, endogenous catecholamines are not involved in cold-induced hypertension. It is possible that the activation of the ET_A_ receptor causes hypertension and cardiac hypertrophy after long-term cold exposure (Fig. 3).

***Cardioprotective effect of cold*** ***adaptation ***

Short-term cold exposure contributes to an increase in the level of catecholamines and is associated with an increase in oxygen demand in humans[36^,^^62^]. In addition, prolonged (seven weeks) cold exposure causes a rise in oxygen consumption in mice[63]. Therefore, it could be hypothesized that adaptation to cold would cause a decrease in cardiac tolerance to IRI. However, we found that continuous cold adaptation (4 °C; 4 weeks) increases the rat heart’s tolerance to ischemia (45 min) and reperfusion (2 h)[34,64]. Our data also indicated that intermittent cold adaptation (4 °C, 8 h/day, 4 weeks) or intermittent cold exposure (4 °C, 1.5 h/day, 4 weeks) had no effect on cardiac tolerance to IRI[34]. A Czech research group found that chronic cold exposure (8 °C, 8 h/day for a week, followed by 4 weeks at 8 °C for 24 h/day) augments cardiac tolerance to ischemia (20 min) and reperfusion (3 h), and this effect persists for at least 14 days[39]. A Russian research group found that the infarct-limiting effect of cold adaptation is not associated with serum cortisol, corticosterone, T_3_, and T_4_ levels[34]. Cold exposure did not affect the appearance of peptic ulcers in the stomach or the involution of the thymus and spleen[34]. Continuous cold exposure induced a 40% increase in adrenal gland hypertrophy. Therefore, chronic cold exposure is not considered a form of stress. Both continuous and intermittent cold exposure cause an increase in brown fat weight, heart weight, and left ventricle weight, which are typical alterations for cold adaptation[65,66]. Tibenska et al. observed that the infarct-reducing effect of adaptation to cold is not accompanied by β_1_-AR expression, PKA, the p-PKA level, and adenylyl cyclase activity[39]. Simultaneously, they found that cold adaptation increased the tolerance of cardiac mitochondria to Ca^2+^ overload, which may indicate the important role of the permeability transition pore (MPT pore) in the cardioprotective effect of cold adaptation[39]. There is evidence that chronic cold exposure (4 °C; 4 weeks) had no effect on the level of autophagy markers (p62, LC3II, and LC3I) in myocardial tissue of sham-operated mice[67]. However, the levels of these markers were altered in mice with abdominal aortic constriction after cold adaptation, indicating an enhancement of autophagy. It can be assumed that autophagy is involved in the cardioprotective effect of cold adaptation. Jankovic and colleagues found that cardiac tolerance to IRI increased with adaptation to hypoxia[^68^], and the specificity of cold adaptation was an increase in oxygen consumption[^69^,^70^]. In cold-adapted mice, oxygen consumption remains increased at room temperature (20 °C)[71]. However, it should be noted that cold exposure (4 °C; 10 days) does not affect heart’s oxygen consumption^[^^72^^]^. Hypoxia-inducible factor-1α decreased in brown adipose tissue of rats after chronic cold exposure (4 ± 1 °C) for 12, 21, and 45 days[73], but not in white adipose tissue of rats after cold adaptation (4 ± 1°C, for 3, 7, 12, 21, and 45 days)[^68^]. Consequently, the molecular mechanism of the cardioprotective effect of cold adaptation must be different from the molecular mechanism of adaptation to hypoxia. Thus, the receptor and signaling mechanism of the infarct-reducing effect of cold adaptation have been still remained unclear. It is not known how cold adaptation affects cardiac contractility during reperfusion and influences programmed cell death during reperfusion (apoptosis, necroptosis, pyroptosis, and ferroptosis). We assume that the same receptors and signaling mechanisms involving in conditioning mediate the protective effect of cold adaptation[^74^,^75^-^77^]. 

***Role of catecholamines in the cardioprotective effect of cold adaptation***

Catecholamines and adrenergic receptors play an important role in cold adaptation[39,^78^]. Preliminary stimulation of β-AR increases cardiac tolerance to IRI (Fig. 3)[39,^79^], and the cardioprotective effect of ischemic preconditioning is associated with the activation of the α_1_-AR[^80^]. It has been also shown that the release of endogenous catecholamines by tyramine prior to CAO increases cardiac resistance to IRI[^81^], and α_1_-AR stimulation mimics the cardioprotective effect of ischemic preconditioning[82]. The cardioprotective and antiarrhythmic effects of the α_1_-AR agonists are mediated via G_i/o_-proteins and associated with the activation of PKC and opening of mitoK_ATP_[82-84]. In addition, the cardioprotective effect of the β-AR agonist isoproterenol is mediated via the activation of PKC-δ[85]. The infarct-reducing effect of isoproterenol has been indicated to be dependent on the stimulation of β_1_-AR[85]. However, there is evidence that the β_1_-AR agonist denopamine, the β_1_-, β_2_-AR agonist isoproterenol, and the β_2_-AR agonist formoterol decrease infarct size and improve cardiac contractility during reperfusion[86]. The antioxidant N-acetylcysteine eliminates the infarct-sparing effect of isoproterenol, but the mitoK_ATP_ channel blocker 5-hydroxydecanoate does not affect the cardioprotective effect of isoproterenol. These facts suggest that ROS are involved in the development of the cardioprotective effect of isoproterenol[86]. The above-mentioned studies indicate that the activation of α_1_-AR, β_1_-AR, and β_2_-AR can increase cardiac tolerance to IRI. Since endogenous catecholamines are involved in the development of cold adaptation, it can be hypothesized that they also contribute to the development of the cardioprotective effect of cold adaptation. Tibenská and colleagues found that the infarct-reducing effect of cold adaptation does not depend on β_1_-AR expression. However, it is probable that other ARs can be involved in the cardioprotective effect of cold adaptation. The same research group also demonstrated that the persisting infarct-limiting effect of chronic cold adaptation mediates via β_2_-AR stimulation[87]^.^

***Role of thyroid hormones in the cardioprotective effect of cold adaptation***

Thyroid hormones play a role in the cardioprotective effect of adapting to cold[66] and stimulate TRs: TRα and TRβ^[^^88^^]^. Data on the role of thyroid hormones in regulating cardiac tolerance to IRI are contradictory. It has been reported that thyrotoxicosis does not affect cardiac resistance to IRI, and hypothyroidism promotes a decrease in infarct size in rats[^89^]. In a study by Jeddi et al., the isolated hearts from hypothyroid rats were subjected to 30 minutes of global ischemia, followed by 120 minutes of reperfusion[90]. They results showed that hypothyroid rats’s hearts were resistant to IRI. In Suarez et al.’s study, hypothyroidism contributed to a decrease in infarct size and reduced the release of lactate dehydrogenase and creatine kinase from the isolated heart. However, it was demonstrated that overexpression of endothelial TRα1 contributes to a 45% decrease in infarct size in mice[91]. In another study, pretreatment with thyroxine (25 μg/100 g/day subcutaneously) for two weeks increased the tolerance of the isolated rat heart to IRI[92]. Moreover, 3,5-Diiodothyropropionic acid, a T_3_ analog that binds to the TRα and TRβ, reduced infarct size and attenuated inflammatory cardiac injury after permanent CAO in mice[93]. In an investigation conducted on the isolated perfused rat heart subjected to IRI, T_3_ reduced infarct size[94]. The inconsistent data on the role of TRs in regulating cardiac tolerance to IRI seems to be linked to the presence of two TR (TRα and TRβ) subtypes. It is possible that the activation of one receptor enhances cardiac resistance to IRI, but stimulation of another TR aggravates IRI cardiac injury. In this regard, the selective TRα and TRβ antagonists could clarify the situation. Since thyroid hormones play an important role in cold adaptation, it can be assumed that they are involved in the infarct-reducing effect of adaptation (Fig. 3).

***Role of ROS in the cardioprotective effect of cold adaptation***

It is well known that ROS are involved in the cardioprotective effect of ischemic pre- and post-conditioning^[^^95^^]^. In a previous study, cold exposure (5°C; 1.5 h; 28 days) had no effect on the diene conjugates and MDA levels in the myocardium of rats. Moreover, catalase and superoxide dismutase activity increased in cardiac tissue of rats[96]. In another study, rats were subjected to cold exposure (5 °C; 5, 10, 15, and 49 days). The results demonstrated that cold exposure had no effect on the MDA level in myocardial tissue, and chronic cold exposure (4 °C; 4 weeks) had no effect on ROS generation in the myocardial tissue of sham-operated mice^[^^67^^]^. Other investigators have shown that cold adaptation (4 °C; 6 h during 14 days) leads to the increased ROS production in myocardial tissue of rats[^97^], and cold-weather field training increases the serum lipid hydroperoxides level in human^[^^98^^]^. In Schmidt et al.’s study, cold adaptation (4 °C; 6 months) promoted an increase in glutathione peroxidase activity in the rat heart without affecting glutathione reductase activity[^99^]. Selman et al. found that cold exposure (8 °C; 18 days) increased catalase activity in myocardial tissue of small mammals (*Microtus agrestis*) without altering superoxide dismutase activity^[^^100^^]^. Emirbekov et al. observed that cold adaptation (-5 °C; 3 h; during 20–25 days) decreased the MDA level in the myocardium and increased total antioxidant activity in the myocardial tissue of rats[^101^]. We found that a free radical scavenger, N-2-mercaptopropionylglycine, abolished the infarct-reducing effect of cold adaptation [unpublished data]. Thus, there is currently no convincing evidence that cold adaptation enhances or inhibits ROS production in animals without I/R cardiac injury or in animals with CAO and reperfusion. 

***Role of*** ***FGF,*** ***TNF-α, M-cholinergic, TRPV1, vasopressin, ghrelin, adenosine, and opioid receptors in the cardioprotective effect of cold adaptation***

FGF is involved in the cardioprotective effect of ischemic pre- and post-conditioning^[^^74^^]^. Cold adaptation (4 °C; 15 days) induced an increase in the plasma FGF21 level in mice^[^^102^^]^; however, some investigators believe that cold adaptation (6 °C; 7 days) decreases the plasma FGF21 level in mice. Thus, the question of the involvement of FGF in the infarct-reducing effect of cold adaptation remains open. It has been reported that TNF-α is involved in the cardioprotective effect of adaptation to hypoxia^[^^103^^]^. There are two TNF-α receptors: TNF-α receptors I (TNFR-I, p55) and II (TNFR-II, p75)^[^^104^^]^. The activation of TNFR-I aggravates IRI^[^^104^^]^, while the stimulation of TNFR-II enhances cardiac tolerance to IRI^[^^103^^]^. Cold adaptation (4 °C; 15 days) induced an increase in the plasma TNF-α concentration in mice^[^^102^^]^. Therefore, it is possible that TNF-α has involvement role in the cardioprotective effect of cold adaptation. It is known that α7nAChR is responsible for the cardioprotective effect of remote postconditioning[105], and the muscarinic receptor has a participation in the infarct-reducing effect of remote preconditioning[106]. Therefore, it can be assumed that these receptors are involved in the cardioprotective effect of cold adaptation. In a study by Manukhin et al., rabbits were exposed daily to severe cold condition (-10 °C; 6 h; 1-30 days), in which an increased sensitivity of blood vessels to acetylcholine was found. The authors suggested that cold adaptation can alter the characteristics of M-cholinergic receptors of blood vessels[107]. In this case, the cardioprotective effect of cold adaptation could be mediated via the activation of M-cholinergic receptors. Gorbunov and colleagues observed the involvement of the TRPV1 channel in the regulation of cardiac resistance to IRI. They have also observed that the TRPV1 activation increases cardiac tolerance to IRI due to calcitonin gene-related peptide release from afferent nerve endings[^108^]. It has been shown that chronic cold exposure (4 °C; 4 weeks) upregulates TRPV1 in the myocardial tissue of mice[67]. However, there are data that cold exposure (4 °C; 5 weeks) downregulates TRPV1 in the murine heart[60]. Consequently, the role of TRPV1 in the infarct-reducing effect of cold adaptation requires further study. Pretreatment with vasopressin has been demonstrated to decrease infarct size in rats[^109^], while chronic cold exposure has been found to increase the plasma level of vasopressin in guinea-pigs[1^10^]. Therefore, vasopressin could be involved in the cardioprotective effect of cold adaptation. There is evidence that an uncharacterized pertussis toxin-insensitive receptor localized in guinea pig cardiomyocytes could play a role in cold adaptation[^111^]. This receptor, which is expressed in myocardial tissue, could be the PPARγ[^112^]. The activation of PPARγ enhances cardiac tolerance to IRI[^113^]. It has been demonstrated that cold exposure (4 ± 1 °C for 1, 3, 7, 12, 21, and 45 days) increases PPARγ expression in the skeletal muscle of rats[^114^]. If an increase in PPARγ expression is observed in myocardial tissue, this will enhance cardiac tolerance to IRI, suggesting the involvement of α7nAChR. In this regard, FGF, TNF-α, M-cholinergic, and PPARγ receptors are found to be involved in the cardioprotective effect of cold adaptation (Fig. 3).

***Role of*** ***protein kinases, NOS, MPT pore, and K***_ATP_*** channels in the cardioprotective effect of cold adaptation***

Chronic cold exposure (4 °C; 4 weeks) had no effect on the phosphorylated AMP-activated protein kinase (p-AMPK), p-mTOR kinase (mammalian target of rapamycin), in the myocardial tissue of sham-operated mice^[^^67^^]^. However, after cold adaptation, the levels of p-AMPK and p-mTOR altered in mice with abdominal aortic constriction. Cold adaptation (4 ± 1 °C; 3, 7, 12, 21, and 45 days) led to an increase in p-AMPKα expression in the white adipose tissue of rats[^68^]. It is well known that these kinases are involved in regulating cardiac tolerance to IRI[^74^^|^]. Therefore, it can be assumed that they are involved in the cardioprotective effect of cold adaptation. The role of other kinases in cold adaptation remains unknown. We established that inducible NOS plays an important role in the infarct-reducing effect of adaptation to chronic hypoxia[^115^]. It has also been demonstrated that cold exposure enhances endothelial NOS expression in the brown adipose tissue of rats[^116^]. However, there is no data on the effect of cold adaptation on NOS expression in the heart. An earlier study has suggested that long-term cold exposure (5 ± 2 °C; 5 weeks) decreases the plasma nitrite and nitrate levels in mice[58]. These data indicate a reduction in NO production after cold adaptation. It has been known that MPT pore closure is involved in the cardioprotective effect of ischemic preconditioning and postconditioning[^74^]. Tibenska and colleagues obtained indirect evidence of the involvement of MPT pore in the cardioprotective effect of cold adaptation in rats[39]. There is evidence that K_ATP _channels are also involved in the cardioprotective effect of pre- and postconditioning[^74^,^75^], as well as adaptation to continuous hypoxia^[^^115^^]^. We found that the K_ATP _channel blocker, glibenclamide, abolished adaptation-induced cardiac tolerance to IRI [unpublished data] (Fig. 1).

**Can angiotensin-II and endothelins increase cardiac tolerance to IRI?**

The cardioprotective effect of angiotensin II during ischemia and reperfusion of the heart has been well-documented^[^^117^^,^^118^^]^. Angiotensin II acts through two receptors: AT_1_R and AT_2_R. Evidence has revealed that the infarct-reducing effect of angiotensin II acts via G protein-independent signaling through the AT_1_ receptor^[^^117^^]^. The cardioprotective effect of stimulating the AT_1_ receptor has been confirmed by Nuñez’s group^[^^118^^-^^120^^]^. However, the blockade of the AT_1_ receptor enhances cardiac tolerance to IRI in mice^[^^121^^]^. In 1996, it was shown that endothelin-1 can mimic ischemic preconditioning against infarction in the isolated rabbit heart through the activation of the ETA receptor and stimulation of PKC^[^^122^^]^. Endothelin-1 protects the isolated rat heart against IRI via the activation of the ET_A_ receptor, stimulation of PKC, and opening of the mitoK_ATP_ channel[^123^]. Recently, it has been shown that endogenous endothelin-1 and the ETA receptor are involved in the cardioprotective effect of remote preconditioning in rats^[^^124^^]^. However, it has been displayed that the selective ET_A_ receptor antagonist BQ123, can also increase cardiac tolerance to reperfusion in rabbits[^125^]. Based on the above-mentioned studies, it is reasonable to hypothesize that endothelin-1 and angiotensin II can play a role in the cardioprotective effect of cold adaptation (Fig. 3). 

## CONCLUSION

Analysis of the published data indicates that cold adaptation increases the incidence of developing hypertension, coronary artery disease, and AMI in human. Moreover, long-term exposure to cold condition causes hypertension, cardiac hypertrophy, and cardiac tolerance to IRI in rats. Cold-induced hypertension is mediated via the activation of aldosterone, AT-1, and ET_A_ receptors. It appears that the activation of AT-1 and ET_A_ receptors causes cardiac hypertrophy after long-term cold exposure. TRPV1, adrenergic, thyroid, MR, ET_A_, AT_1_, PPARγ, α7nAChR. FGF, TNF-α, and M-cholinergic receptors could be involved in the cardioprotective effect of cold adaptation. It is assumed that antioxidants, protein kinases, MPT pore, and K_ATP_ channels contribute to the development of cold adaptation, which triggers cardiac tolerance to IRI. 

## DECLARATIONS

### Acknowledgments

The authors did not use artificial intelligence (AI)-assisted technologies in the production of submitted work.

### Ethical approval

Not applicable.

### Consent to participate

Not applicable.

### Consent for publication

All authors reviewed the results and approved the final version of the manuscript.

### Authors’ contributions

NSV: contributed to the acquisition, analysis, and interpretation of data, as well as to the conception**,** writing, and typing of the article, and preparation for printing; SVP, NVN, and IMP: contributed to the acquisition, analysis, or interpretation of data, as well as to the conception or design. NRP, VVK, EAT, and EVS: revised the manuscript critically. LNM: devised the project, the main conceptual ideas of the article, final approval of the content for publication of this manuscript.

### Data availability

The data supporting the findings of this study are within the manuscript. 

### Competing interests

The authors declare that they have no competing interests. 

### Funding

This article was prepared with the support of the Ministry of Science and High Education of the Russian Federation [the state assignment no. 122020300042-4].

### Supplementary information

The online version does not contain supplementary material.
